# The C-terminal region of the plasmid partitioning protein TubY is a tetramer that can bind membranes and DNA

**DOI:** 10.1074/jbc.RA120.014705

**Published:** 2020-10-22

**Authors:** Ikuko Hayashi

**Affiliations:** Department of Medical Life Science, Yokohama City University, Tsurumi, Yokohama, Kanagawa, Japan

**Keywords:** TubZ, crystal structure, bacteria, plasmid partitioning, DNA segregation, segrosome, MerR, amphipathic helix, plasmid, DNA binding protein, cytoskeleton, plasmid partioning

## Abstract

Bacterial low-copy-number plasmids require partition (*par*) systems to ensure their stable inheritance by daughter cells. In general, these systems consist of three components: a centromeric DNA sequence, a centromere-binding protein and a nucleotide hydrolase that polymerizes and functions as a motor. Type III systems, however, segregate plasmids using three proteins: the FtsZ/tubulin-like GTPase TubZ, the centromere-binding protein TubR and the MerR-like transcriptional regulator TubY. Although the TubZ filament is sufficient to transport the TubR-centromere complex *in vitro*, TubY is still necessary for the stable maintenance of the plasmid. TubY contains an N-terminal DNA-binding helix-turn-helix motif and a C-terminal coiled-coil followed by a cluster of lysine residues. This study determined the crystal structure of the C-terminal domain of TubY from the *Bacillus cereus* pXO1-like plasmid and showed that it forms a tetrameric parallel four-helix bundle that differs from the typical MerR family proteins with a dimeric anti-parallel coiled-coil. Biochemical analyses revealed that the C-terminal tail with the conserved lysine cluster helps TubY to stably associate with the TubR-centromere complex as well as to nonspecifically bind DNA. Furthermore, this C-terminal tail forms an amphipathic helix in the presence of lipids but must oligomerize to localize the protein to the membrane *in vivo*. Taken together, these data suggest that TubY is a component of the nucleoprotein complex within the partitioning machinery, and that lipid membranes act as mediators of type III systems.

Accurate DNA segregation is essential for transmission of genetic information to daughter cells. The well-studied process of eukaryotic DNA segregation involves the mitotic spindle and kinetochores, which are composed of multiple protein complexes. By contrast, much less is known about prokaryotic DNA segregation.

Partition systems (*par*) for low-copy-number plasmids are thought to be the best model for studying bacterial DNA segregation, as only three components are required: a centromere DNA site, a centromere-binding protein (CBP) and an ATP or GTP hydrolase (NTPase; 1, 2). In general, *par* systems are classified into three types based on the nature of the NTPases that provide the driving force for plasmid segregation. Type I and II partition systems employ a Walker-type ATPase ParA and an actin-like ATPase ParM, respectively, whereas type III systems use an FtsZ/tubulin-like GTPase TubZ. The behaviors of these noncanonical motor proteins are distinct from one another in the cell, implying that the molecular mechanisms of the three systems are dissimilar (2, 3). Plasmids are delivered to daughter cells by these NTPases, whereas the CBPs function as adaptors between the centromere and NTPases. Unlike the NTPases, the sequence homology of the CBPs is fairly low even within the same partition system (4). Hence, the centromere sequences are not conserved but consist mostly of multiple tandem repeats. These repeats contribute to formation of the segrosome, a higher-order nucleoprotein complex containing the CBPs and centromere (reviewed in ref. 5). The segrosome activates the NTPases to achieve partitioning of the plasmid. The type I ATPase ParA nonspecifically associates with nucleoid DNA, and when the segrosome interacts with the ParA on the nucleoid, it stimulates the ATPase activity of ParA, leading to dissociation of ParA from the nucleoid (6). Subsequently the segrosome diffuses and associates with neighboring ParA, which results in a directional movement of the plasmid. In type II systems, the ParM ATPase forms a bipolar spindle whose ends are stabilized by binding to the segrosome and polymerization of ParM forces the segrosome to the cell poles, causing the plasmids to segregate to the daughter cells (7–9). The segrosome of type III systems associates with the minus-ends of TubZ filaments and is pulled by filaments while they treadmill (10).

In all segregation systems, three partitioning factors of the centromere and the proteins are coded within a *par* operon, but in some cases, proteins encoded outside *par* are required for efficient partition. For instance, the centromere region of the P1 plasmid in the type I system contains the binding site for IHF (integration host factor) as well as the CBP (11, 12). IHF can bend DNA by ∼180°, which allows the CBP to associate with the centromere to form a segrosome (13, 14). Another example is TubY from type III partition systems. TubY is a DNA-binding protein with a putative helix-turn-helix (HTH) motif that functions as a transcriptional activator of the *tubRZ* operon (15). More importantly, TubY modulates the interaction between the segrosome and the TubZ filaments (16); however, it remains unclear how TubY is involved in plasmid segregation.

The type III *par* system was first identified in virulent *Bacillus* species (17, 18). The centromere *tubC* is localized upstream of the tandemly arranged *tubR* and *tubZ* genes and consists of several direct or inverted repeats, providing multiple binding sites for TubR, the CBP (16, 18–21). TubR binding leads to segrosome formation as well as transcriptional repression of *tubRZ*. TubY is not encoded inside the *tubRZ* operon, but its locus is in the same vicinity (16). Stable maintenance of the plasmid requires TubY, which strongly suggests that *tubY and tubRZ* form a regulon for plasmid partitioning (15). Notably in this regard, *tubY* from virulent *Bacillus* species is located upstream of *tubRZ*, whereas *tubY* from *Clostridium botulinum* is downstream of *tubRZ*, and the transcription direction of *tubY* is not conserved among type III systems (16). These observations raise the question of how much diversity, both structurally and functionally, type III systems possess.

Here, I report the crystal structure of the TubY C-terminal coiled-coil domain of the pXO1-like plasmid pBc10987 from *Bacillus cereus*. The structure contains a tetrameric four-helix bundle that enables the N-terminal HTH motif to bind DNA. In addition, I found that the amphipathic C-terminal tail of TubY contributes not only to nonspecific DNA binding but also to association with lipid membranes. These data suggest that TubY may function as a modulator of segrosome formation and localization in the cell.

## Results

### TubY binds upstream of the tubRZ operon in pBc10987

**Figure 1.**
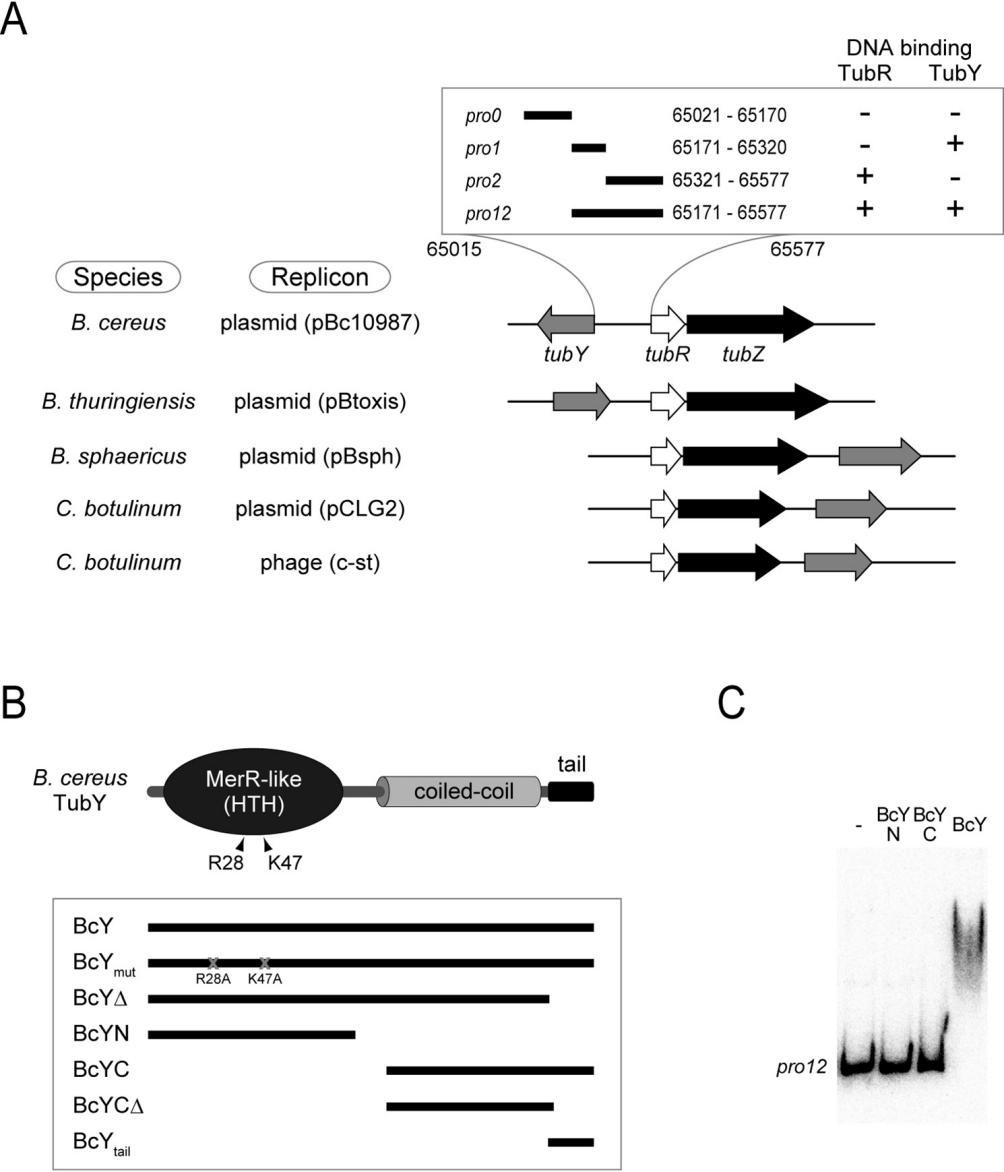
**The tubYRZ regulon.** *A*, Gene organization of the *tubYRZ* regulon in the plasmids from different *Bacillus* species (pBc10987 from *Bacillus cereus,* pBtoxis from *Bacillus thuringiensis* and pBsph from *Bacillus sphaericus*), along with the regulons in the pCLG2 plasmid and prophage c-st from *Clostridium botulinum*. Type III partitioning systems containing *tubR*, *tubZ* and *tubY* are listed. Transcription directions of *tubY* (gray), *tubR* (white) and *tubZ* (*black*) are shown as arrows. DNA fragments used in this study are shown in the box. Binding of TubR and TubY, determined by the pulldown assay, is indicated. *B*, Domain organization of *Bacillus cereus* TubY. Mutations critical for DNA binding are labeled. Constructs used in this study are shown in the box. *C*, EMSA analysis of BcY and its mutants (BcYN and BcYC) binding to the *pro12* region. Protein concentration is 1 μm. Reactions were analyzed by electrophoresis using a 6% polyacrylamide gels.

TubY, a putative MerR family transcriptional regulator, is encoded upstream of the *tubRZ* operon in pBc10987 (Fig. 1*A*). In type III *par* systems, although *tubY* genes are located in the vicinity of *tubRZ*, they are not likely to be transcribed as part of the same transcriptional unit (15, 16). Because the *tubY* promoter is flanked by *tubRZ*, I examined the binding of TubR or TubY of pBc10987 to the region of DNA between *tubY* and *tubR*. DNA fragments from the promoter regions were radioactively labeled and examined for binding of His-tag fused TubR or TubY by pulldown assays. TubY preferentially bound to the *pro1* region (65171–65320 nt) carrying the putative promoter -35 and -10 elements of *tubRZ*, but not to the TubR-binding region *pro2* (65321–65577; 21).

Full-length TubY from *B. cereus* (BcY) was mostly expressed as an insoluble protein, similar to the one previously reported for *C. botulinum* TubY (16). Therefore, denatured BcY was refolded and purified to homogeneity. During purification, BcY was proteolytically sensitive: specifically, it was degraded into two peptides, indicating that it possesses structurally flexible regions. To identify the domain structure of BcY, the digested fragments were analyzed by MS and determined to be BcYN and BcYCΔ (Fig. 1*B*). Sequence analyses revealed that BcYN and BcYCΔ contain the HTH motif and coiled-coil domains, respectively. The conserved C-terminal tail was susceptible to degradation, presumably because of the intrinsic flexibility of the clusters of lysine and phenylalanine residues (see below). In analysis of DNA binding by BcYN and BcYCΔ with the tail (BcYC) using electrophoretic mobility shift assays (EMSA), only BcY bound *pro12* (65171–65577 nt), indicating that both domains are required for DNA binding (Fig. 1*C*).

### BcY is a tetramer

**Figure 2.**
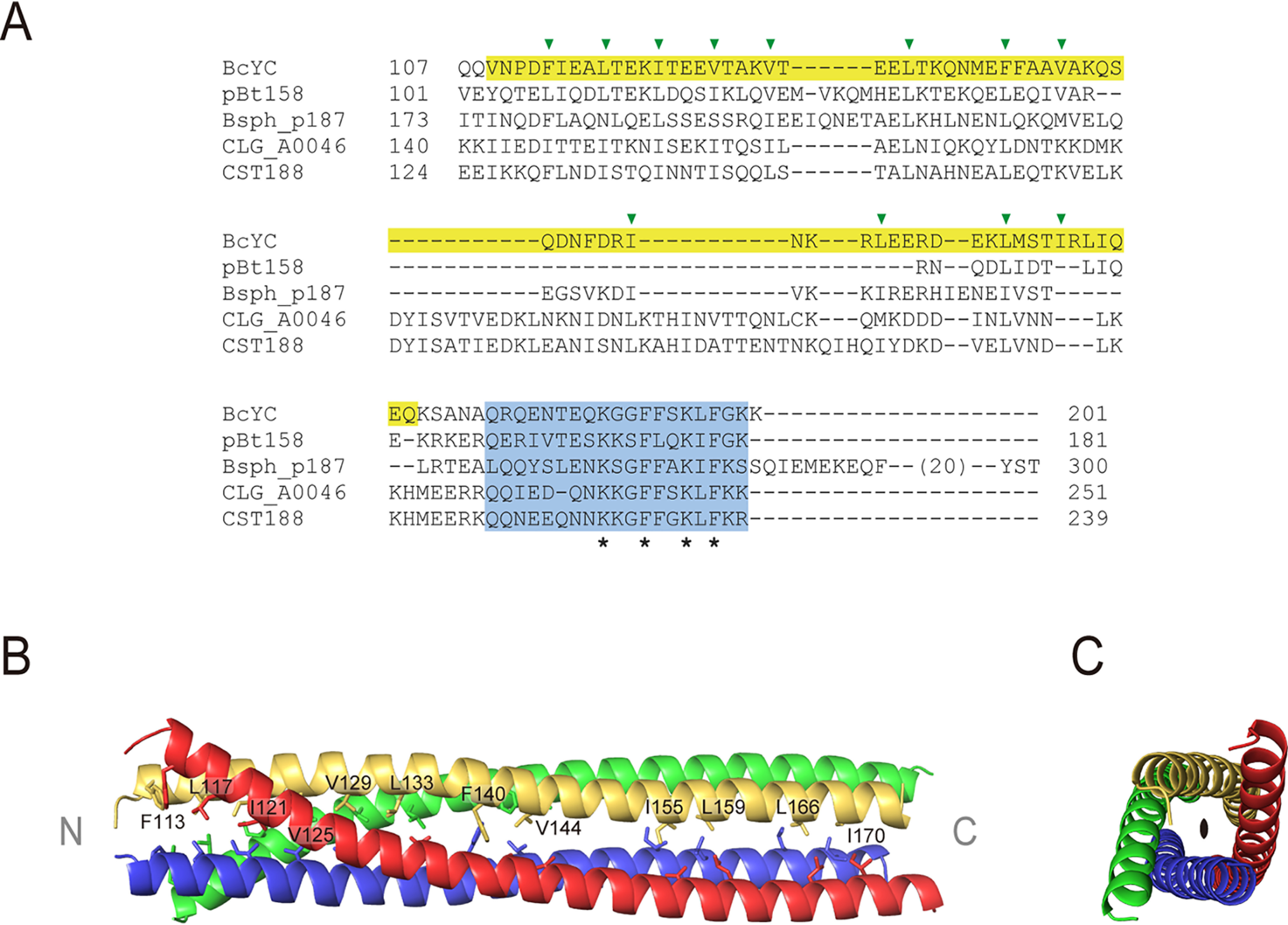
**Crystal structure of the tetramerization domain of BcY.** *A*, Sequence alignment of the C-terminal domains in TubY listed in Fig. 1*A*: BcYC (NCBI Reference Sequence ID: WP_000564666; pBt158 from pBtoxis (WP_086402658); Bsph_p187 from pBsph (WP_069511974); and CLG_A0046 (ACT33709) and CST188 (YP_398618) from pCLG2 and c-st, respectively. The α-helix observed in the crystal structure is highlighted in yellow. The C-terminal amphipathic tail is shown in cyan. Residues involved in the hydrophobic core are indicated by green arrows. Conserved residues in the C-terminal tail are indicated by asterisks. *B*, Tetramerization domain of BcY. Residues forming the hydrophobic core are shown as sticks and labeled. Each monomer is shown in a different color. The N and C termini are labeled. *C*, Top view of the tetramerization domain. The orientation in (*C*) is related to that in (*B*) by a 90° rotation about a vertical axis. The 2-fold axis is indicated.

Although the primary sequence of the C terminus is not tightly conserved among the TubY proteins encoded by the plasmids or phage, it is predicted to form a coiled-coil (16; Fig. 2*A*). I determined the crystal structure of BcYCΔ (Fig. 1*B*, Fig. 2*B* and Table 1). The asymmetric unit contains four BcYCΔ monomers, which forms a tetrameric four-helix bundle with dimensions 90 Å × 20 Å × 20 Å. These four monomers are parallel and related by a pseudo-2-fold axis, and the root-mean-square deviation (rmsd) of Cα atoms (residues 112–174 of chain A and B *versus* C and D) is 0.37 Å (Fig. 2*C*). The BcYCΔ structure reveals that the hydrophobic core of the four-helix bundle is mostly aliphatic (Fig. 2*A*, *B*). Because of sequence similarity at the N terminus, TubY is a member of the MerR family (16). The MerR family is a group of metal-dependent transcriptional regulators which typically have a HTH motif at the N terminus followed by a coiled-coil region (23; Fig. S1). The coiled-coil region of the MerR proteins forms an anti-parallel dimer that functions as a metal sensing domain: metals are coordinated by conserved histidine or cysteine residues, which induce a conformational change and activate transcription. Another MerR family protein, TnrA, is a fairly small dimeric protein with a unique winged-HTH structure (24). Regarding BcY, BcYN is monomeric (Fig. S2) and incapable of binding to DNA (Fig. 1*C*), whereas BcYC is a tetrameric parallel coiled-coil with no cysteine or histidine residues. On the basis of these findings, I predict that TubY activates the transcription of *tubRZ* in a different manner than MerR or TnrA.

**Table 1:** **Crystallographic statistics**

**Data Collection**	
	BcYCΔ (Se-MAD)
Beamline	PF-NW12A
Space group	*P*1
Unit cell dimensions, Å, °	*a* = 27.1, *b* = 40.3, *c* = 80.7 α = 102.0, β = 94.2, γ = 98.5
Wavelength, Å	0.9791
Data range, Å	30 – 2.6
Completeness*^c^*, %	98.0 (95.1)
Redundancy^c^	3.9 (3.6)
*I*/σ*(I)*^c^	27.2 (4.3)
*R*_merge_*^a^*^,^*^c^*	0.064 (0.330)
**Refinement**	
Resolution range, Å	26.2 – 2.6
No. reflections	9865
*R*_cryst_ (*R*_free_)*^b^*^,c^	0.224 (0.277)
RMSD: bond length, Å	0.0075
RMSD: bond angle, °	1.52
B factors, Å^2^: protein	63.9
B factors, Å^2^: water	63.5
PDB ID code	7C7Y

*^a^*R_merge_ = ∑ |I_obs_ − |/∑ I_obs_, where I_obs_ is the intensity measurement and is the mean intensity for multiply recorded reflections (22).

*^b^R_cryst_* and *R_free_ = ∑ ‖Fobs| - |Fcalc‖/|Fobs|* for reflections in the working and test sets, respectively. The R-free value was calculated using a randomly selected 5% of the data set that was omitted through all stages of refinement.

*^c^*Numbers in parentheses refer to statistics for the highest shell of data.

### BcY associates with the TubR-centromere complex

To analyze the contribution of each domain to DNA binding, I constructed two BcY mutants: BcY_mut_, which possesses two mutations (R28A and K47A) in the HTH motif, and BcYΔ, which lacks the C-terminal tail (Fig. 1*B*). Curiously, both mutants were expressed in soluble form in *E. coli*, but were purified in the same manner as WT BcY. CD (CD) spectroscopy confirmed that these refolded proteins, as well as soluble BcY_mut_ and BcYΔ, were correctly folded (Fig. S3).

**Figure 3.**
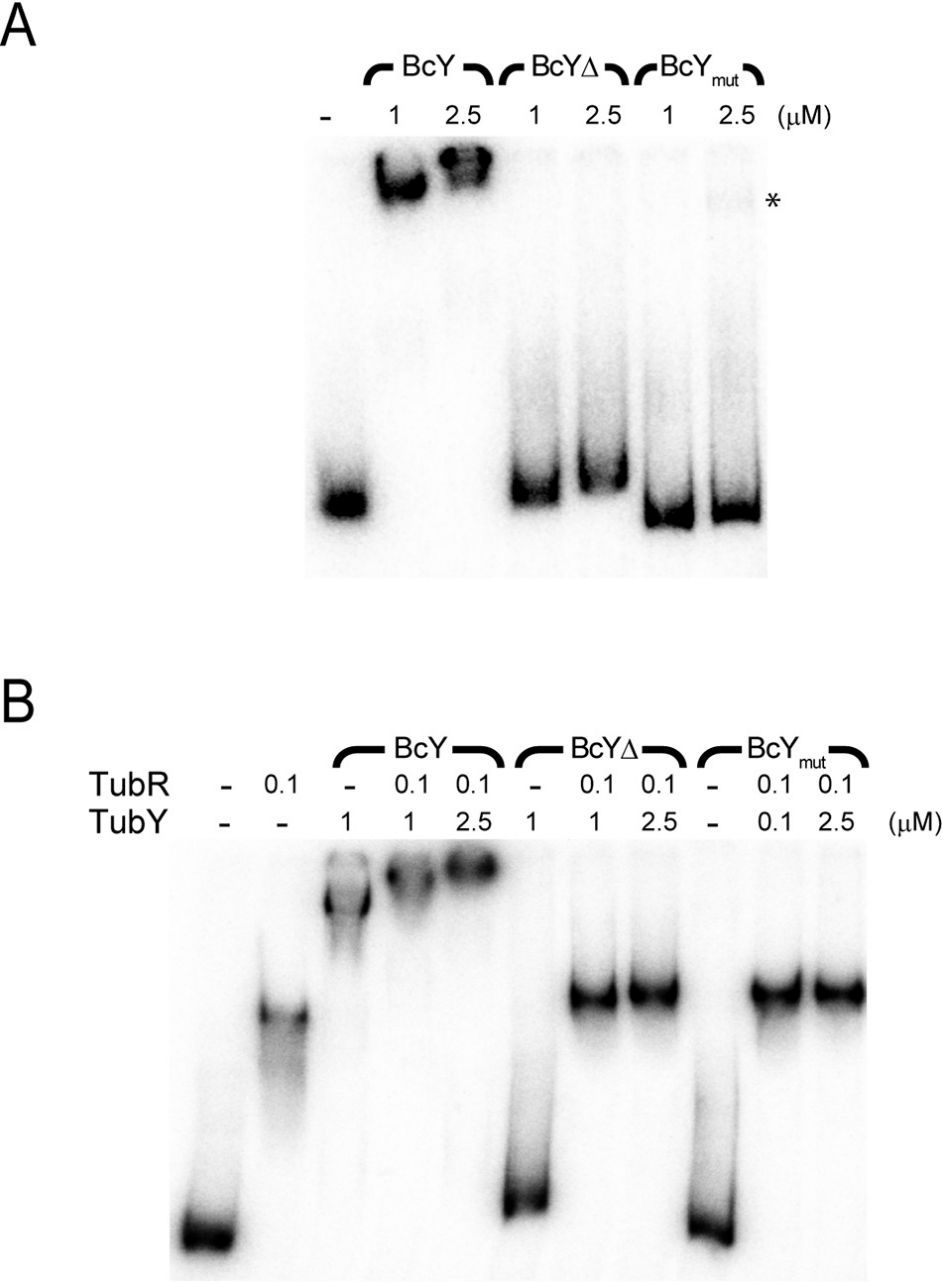
**DNA-binding analysis of BcY and its mutants.** *A*, EMSA analysis of binding of BcY and its mutants BcYΔ and BcY_mut_ to the *pro12* region. The amounts of BcY (in µM) are indicated above the lanes. Asterisk indicates very weak binding of BcY_mut_ to *pro12*. Reactions were analyzed by electrophoresis on 4% polyacrylamide gels. *B*, EMSA analysis of BcY and TubR with *pro12*.

EMSA analysis of BcY with the *pro12* DNA fragment revealed complex formation, consistent with the results of the pulldown assay (Fig. 1*A* and Fig. 3*A*). The R28 and K47 residues are the putative DNA-binding sites predicted from sequence alignment of the MerR family proteins with BcYN (Fig. S1). These residues were simultaneously mutated to alanine. As predicted, the mutations abolished the DNA-binding activity of BcY, indicating that BcYN is critical for DNA binding but must oligomerize with BcYC to form a protein-DNA complex (Fig. 1*C* and Fig. 3*A*). When BcYΔ was mixed with *pro12*, it retarded the mobility of *pro12*, but the complex migrated faster than the BcY-*pro12* complex, implying that the tail region of BcY contributes a nonspecific interaction with DNA (Fig. 3*A*).

Next, to examine binding of BcY and TubR to *pro12*, TubR was preincubated with *pro12* and then BcY was added (Fig. 3*B*). EMSA analysis revealed that the TubR-*pro12* complex band was further supershifted in the presence of BcY, indicating that TubR and BcY bind their own binding sites in *pro12*. When BcY_mut_ or BcYΔ was added to the mixture of *pro12* and TubR, the bands were slightly retarded relative to TubR alone, implying that the BcY mutants may associate with the centromere-bound TubR. These EMSA analyses demonstrate that both the HTH motif and the C-terminal tail of BcY are required for stable segrosome formation.

**Figure 4.**
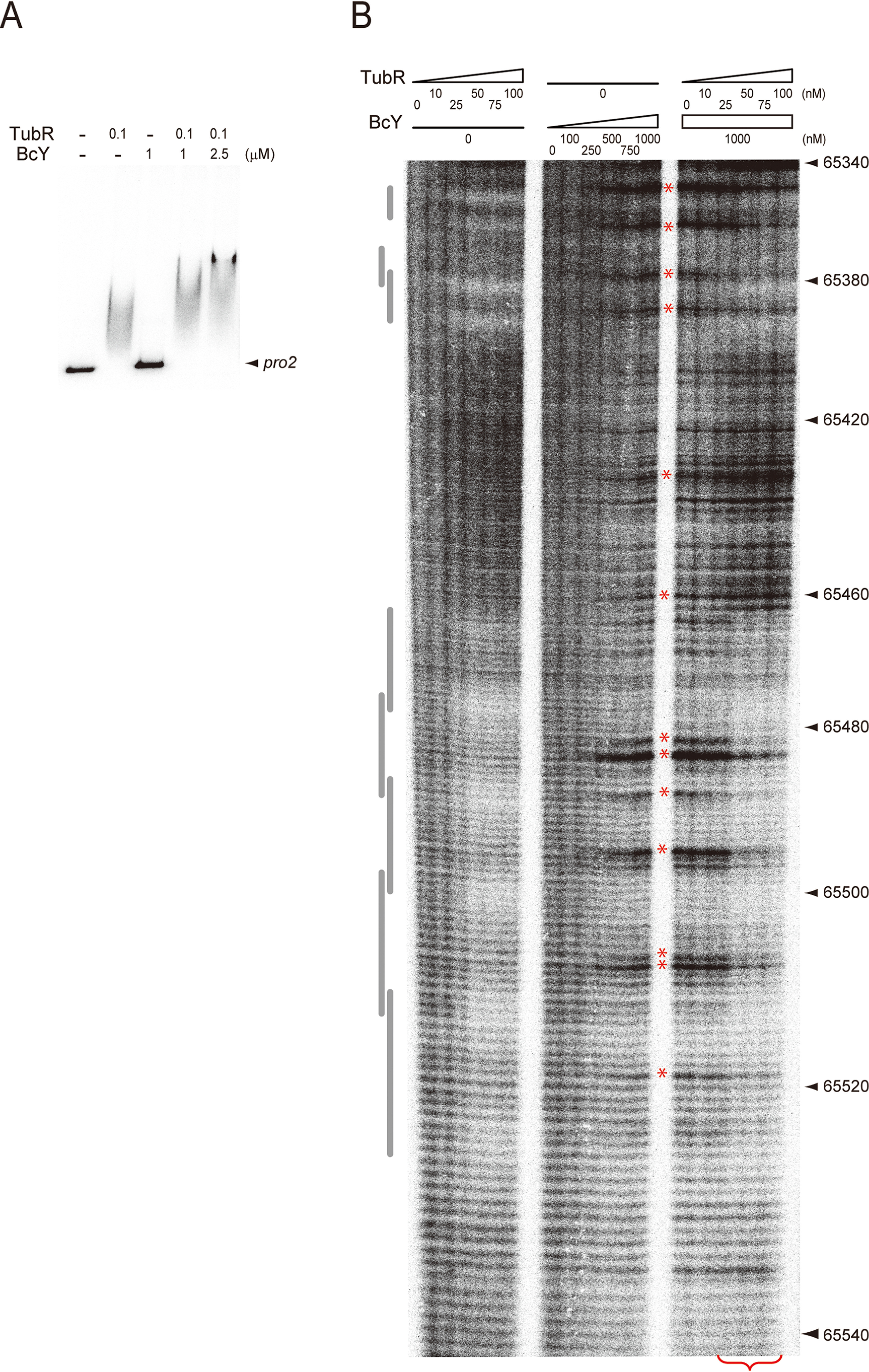
**Centromere binding of TubR and BcY.** *A*, EMSA analysis of TubR and BcY binding to the *pro2* region. TubR binds *pro2*, whereas BcY does not. Addition of both TubR and BcY results in a supershifted *pro2* band, indicating that BcY binds to the centromere and TubR complex. Reactions were analyzed by electrophoresis using a 6% polyacrylamide gel. *B*, Hydroxyl radical footprinting analysis of the *tubRZ* promoter region. The amounts of TubR and BcY (in nm) are indicated above the lane. The numbers on the right-hand side show the location on the pBc10987 plasmid. Gray bars on the left-hand side indicate the regions containing the TubR-binding sites (21). Hyper-sensitive sites are marked by red asterisks. At higher concentrations of TubR (> 50 nm; *red* bracket), BcY protects the TubR-binding region (65460–65530 nt).

TubR and BcY binding to *pro2* was then examined whether BcY can associate with TubR in complex with *pro2* which lacks BcY-binding sites (Fig. 1*A* and Fig. 4*A*). Although BcY did not bind *pro2*, the TubR-*pro2* complex migrated slower in the presence of BcY compared with TubR-*pro2* alone, which suggests that BcY is involved in segrosome formation. Hydroxyl radical footprinting analysis of *pro2* supported these results (Fig. 4*B* and Fig. S4): BcY generated hypersensitive patterns across multiple sites, indicating that BcY nonspecifically interacts with DNA and might influence DNA structure. In the presence of both TubR and BcY, the footprinting patterns showed that the TubR-binding region was highly protected by BcY at TubR concentrations above 50 nm. Thus, TubR and BcY form a supramolecular complex at the centromeric DNA site, and BcY association is presumably achieved via the BcYCΔ region because the BcY mutants, BcY_mut_ and BcYΔ, only weakly associate with the segrosome (Fig. 3*B*).

### BcY binds lipid membranes in vitro

**Figure 5.**
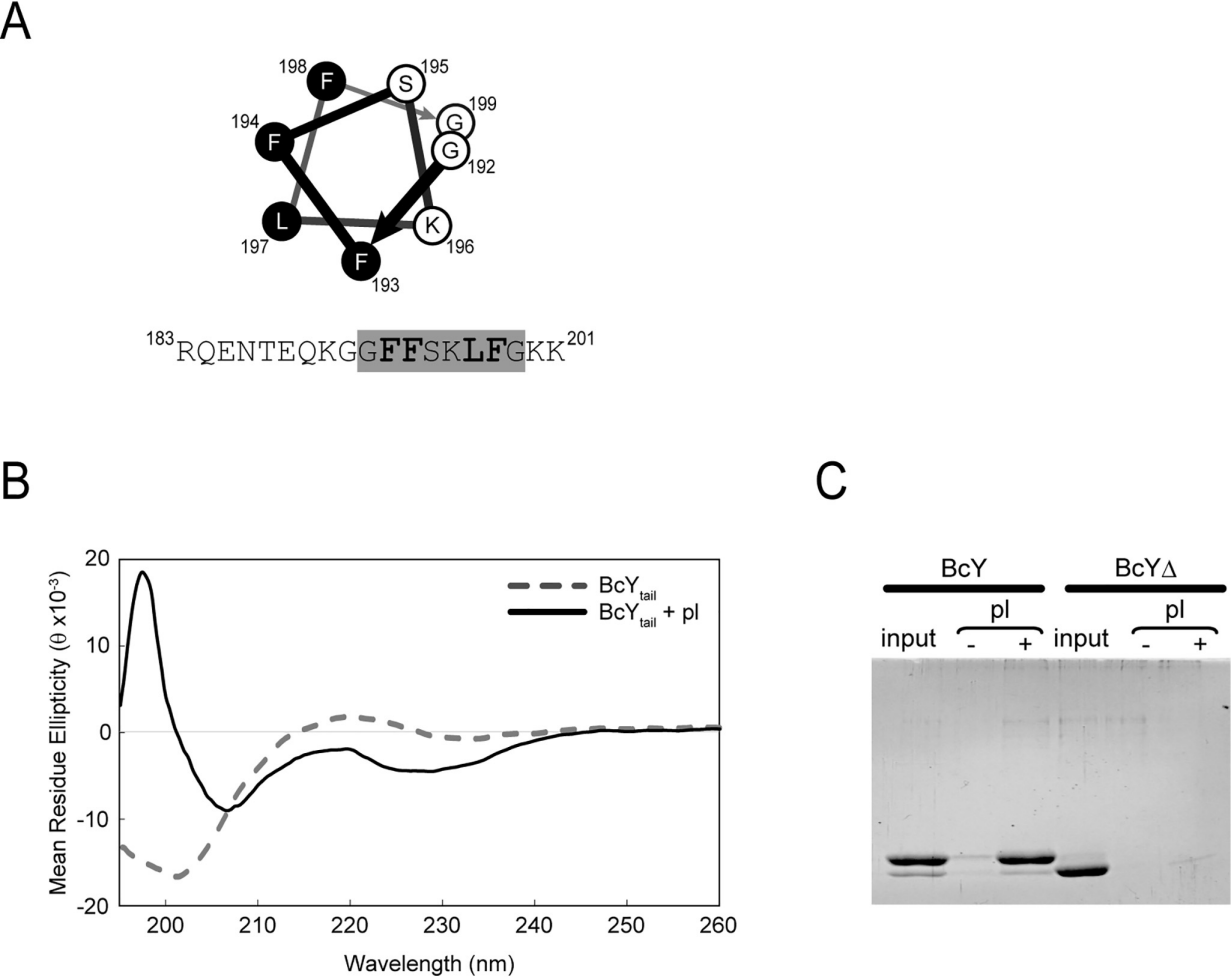
**BcY C-terminal tail binds phospholipids.** *A*, Helical wheel representation of the amphipathic helix in BcY_tail_. Residue numbers are labeled. Hydrophobic residues are highlighted in black. The peptide sequence used in the experiment is given at the bottom: the amphipathic helix region shown in the helical wheel is highlighted in gray (residues 192–199). Hydrophobic residues are shown in a larger font size. *B*, Far-UV circular dichroic spectra of the BcY_tail_ peptide in the absence (dotted line) or presence (*solid line*) of phospholipids (pl). *C*, Co-sedimentation of BcY and BcYΔ with phospholipids. BcY co-sediments with phospholipid vesicles, whereas BcYΔ does not.

The C-terminal tail of the recombinant BcY caused low solubility of the protein. Sequence analysis reveals that this C-terminal tail is conserved not only in TubY of the plasmids or phages from virulent *Bacillus* species or *Clostridium botulinum*, but also in chromosomal TubY from some *Clostridia* (Fig. 2*A* and Fig. S5). This amphipathic tail is a characteristic of many peripheral membrane proteins, including MinD, MreB and SepF (25–28). Analysis of the C-terminal sequence of BcY (BcY_tail_) using the amphipathic helix prediction software AmphipaSeek indicated that the very C-terminal end is likely to form a helix (Fig. 5*A*; 29). Such amphipathic helical structures are thought to interact with lipids directly: the residues on the hydrophobic surface of the helix insert into the lipid bilayer, whereas the cationic residues on the polar surface associate with the head groups of anionic phospholipids.

A synthetic peptide of BcY_tail_ was examined for direct binding to lipid membranes by CD spectroscopy (Fig. 1*B* and Fig. 5*B*). In the absence of lipids, the BcY_tail_ peptide yielded a spectrum with a single minimum at 200 nm, typical of an unstructured peptide and consistent with previous studies of MinD (25). When lipid membranes were mixed with the peptide, significant spectral changes were observed, with a minimum of 206 nm and peak intensity at 228 nm. This spectral change indicates that the BcY_tail_ peptide interacts directly with lipid membranes to yield an α-helical conformation.

To determine whether BcY_tail_ is critical for lipid binding, lipid pelleting assays were performed with BcY or BcYΔ (Fig. 5*C*). BcY co-sedimented with phospholipids, whereas BcYΔ did not, confirming that the BcY_tail_ region is required for interaction with lipid membranes.

### The BcY C terminus is a membrane targeting domain

**Figure 6.**
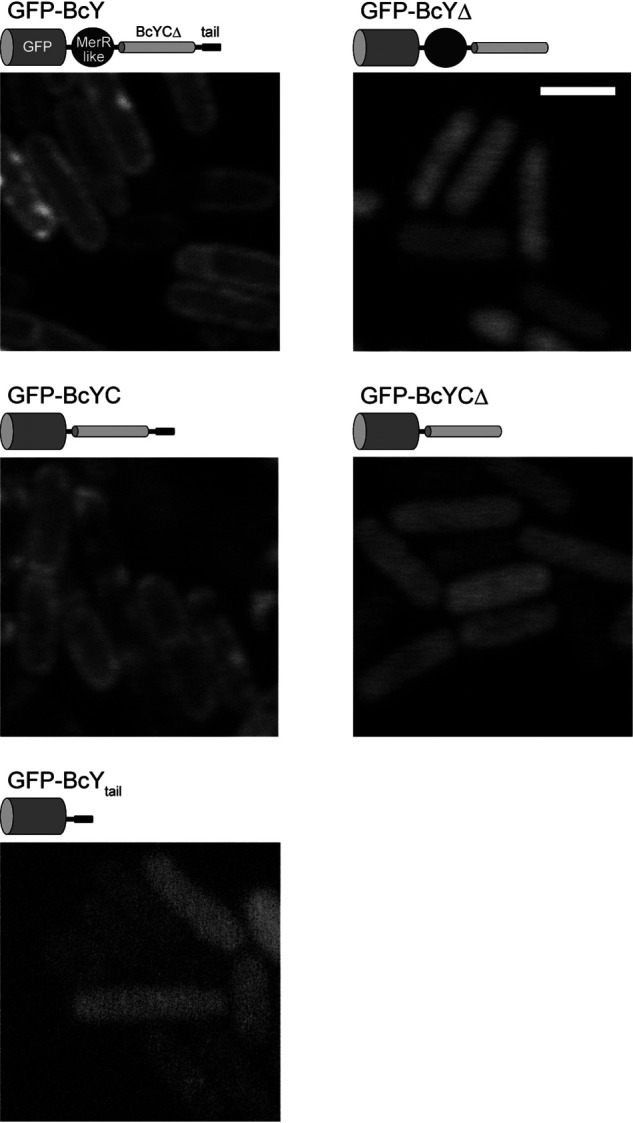
**BcY localization in *B. subtilis* cells.** Fluorescence micrographs of BcY and its mutants (BcYΔ, BcYC, BcYCΔ, BcY_tail_) fused to GFP in *B. subtilis* cells. Cartoons of each construct are shown above the micrographs. Scale bar, 2 μm.

To investigate further whether the C-terminal domain of BcY acts as a membrane targeting motif, BcY and its mutants were fused to the C terminus of GFP and their localization was analyzed *in vivo* (Fig. 6). These experiments were performed in *Bacillus subtilis* because transformation of *B. cereus* cells was not successful. As expected, GFP-BcY exhibited its characteristic distribution around the cell periphery, suggesting that BcY associates with membranes. A similar peripheral localization pattern was observed when GFP-BcYC was introduced, which indicated that the HTH motif is not required for the membrane localization.

Next, to determine the importance of the amphipathic tail in membrane localization of BcY, BcYΔ or BcYCΔ fused to GFP was analyzed. Removal of the C-terminal 19 residues abolished the peripheral localization pattern, and the mutants were uniformly distributed in the cytoplasm. These results suggest that the BcY_tail_ region is essential for membrane localization of BcY. In addition, BcY_tail_ was fused to GFP to investigate whether the BcY_tail_ peptide itself is capable of targeting the cell membrane. GFP-BcY_tail_ was not localized to the cell periphery, indicating that a single BcY_tail_ peptide is not sufficient for the membrane attachment, and that tetramerization by the BcYCΔ region increases the affinity of BcY_tail_ for lipid membranes.

## Discussion

TubY, a putative MerR family protein, is considered to be an integral element of plasmid partitioning in type III *par* systems. This study shows that *B. cereus* TubY is a novel membrane-binding transcription factor and segrosome component by binding to the TubR-centromere complex, suggesting that the cell membrane is involved in the process of type III systems.

The crystal structure of the BcY C terminus revealed that the coiled-coil region is a parallel tetramerization domain, demonstrating that BcY is a unique tetrameric MerR-like transcriptional regulator. The results of this study show that oligomerization enables efficient binding of BcY to DNA. Moreover, the C-terminal tail, which contains basic residues, promotes the interaction with DNA nonspecifically and may change the DNA conformation, as suggested by the footprinting experiment.

The domain organization and DNA-binding function of BcY are analogous to those of tumor suppressor p53. p53 is a tetrameric transcription factor in which an anti-parallel α-helical tetramerization domain is flanked by two distinct DNA-binding regions (reviewed in ref. 30). The central domain binds DNA in a sequence-specific manner, whereas the C-terminal region is a short, unstructured tail of lysine residues that nonspecifically interacts with DNA and can slide along DNA when it is linked to the tetramerization domain. Given the analogy between p53 and BcY, the central DNA-binding domain of p53 corresponds to BcYN which specifically binds *pro1*. Although the current study has not demonstrated the sliding ability of BcY along DNA, the data indicate that several lysine residues in the flexible amphipathic tail of BcY are responsible for nonspecific DNA binding. The lysine-rich C-terminal tail of p53 is also responsible for the recruitment of protein cofactors and can adopt a helical conformation when interacting with DNA or some of the cofactors (30–32). Although shorter than the p53 tail, BcY_tail_ may also allow BcY to engage various partners in different ways to regulate transcription and plasmid partitioning by changing the conformation of BcY_tail_.

Previously, *tubY* and *tubRZ* were shown to form a regulon whose gene products work cooperatively (15, 16). The EMSA analyses in this study showed that BcY associates with the TubR-DNA complex to form a supramolecular structure, implying that BcY is a component of the segrosome. Although the stoichiometry and affinity between the segrosome and BcY remain undetermined, full-length BcY is required for the stable complex. In particular, the BcYCΔ domain seems critical for complex assembly, in which BcY_tail_ plays a supportive role. It should be noted that the cooperativity between TubR and TubY varies among species: *tubC* of pBsph is composed of three blocks of repeated sequences, and when TubY binds its recognition site, TubR is somehow removed from one of the blocks (15). The association of TubY with TubR and *tubC* may induce structural rearrangement of the ternary complex. Further molecular analysis is required to dissect the process of segrosome formation involving BcY.

Structural analyses of TubR-*tubC* complexes have been performed for four species (19, 21, 33). The results of these studies revealed that the nucleoprotein complexes from pBtoxis and pBc10987 have an extended filament structure (19, 21), whereas the others possess a rather rigid ring form (19, 33), indicating that the structural rearrangement might occur to function as a segrosome. TubR-*tubC* of pBtoxis is sufficient for tracking the minus-end of the TubZ filament, but it remains to be determined how the filaments drop off the plasmid to the daughter cells (10). TubR-*tubC* of pBtoxis has been shown to interact with the flexible C-terminal tail of TubZ (34). The TubZ tail of pBc10987 and pBtoxis, which is critical for TubZ assembly, possesses clusters of basic residues (34–36) and is somewhat similar to the amphipathic tail of BcY. In this study, I demonstrated that BcY directly associates with TubR-*tubC*. In type III plasmid partitioning, TubR-*tubC,* which is pulled by the TubZ filament, may abandon the filament and preferentially associate with TubY. Previous analysis of *C. botulinum* TubY showed that TubY, together with TubR-*tubC*, induces disassembly of the TubZ polymers (16). If this is a common mechanism in type III systems, the observation implies either that the quaternary complex between TubZ, TubY and TubR-*tubC* is highly unstable or that the binding site of TubY does not coincide with that of the TubZ filament in TubR-*tubC*. Both possibilities result in unloading of the plasmid, and thus depolymerization of the filaments, as TubR-*tubC* stabilizes the minus-end of the filament (10). Ultimately, the C-terminal tail of the cytoskeletal proteins may use a tail-mimicking mechanism that enables interplay between the filament and the associated proteins, as observed between tubulin and EB1 at microtubule plus-ends (37).

BcY_tail_ interacts with lipid membranes as well. The amphipathic helix is seen in many peripheral membrane proteins, but barely found in transcriptional regulators. One of the exceptions is the yeast transcriptional repressor Opi1, which uses the amphipathic helix to occasionally target lipid membranes by altering its affinity for phosphatidic acid (38, 39). The amphipathic helices are suggested to sense membrane curvature or recognize specific lipids (38, 40, 41). In the case of TubY, the sequence analysis indicates that the amphipathic tail is conserved in TubY (Fig. 2*A* and Fig. S5). Although exact functions of the amphipathic tail are unknown, TubY may play a critical role in the localization of the segrosome and TubZ filaments. It should be noted that, in some *Clostridia,* TubY and TubZ but not TubR are encoded on the chromosome (16). Because chromosome segregation of *Clostridium* seems to rely on Soj (ParA) and Spo0J (ParB), TubZ and TubY may not be involved in DNA segregation (16, 42). In either case, because the chromosome-encoded TubY proteins possess a putative amphipathic helix (Fig. S5), TubY presumably functions as a modulator of TubZ filaments.

**Figure 7.**
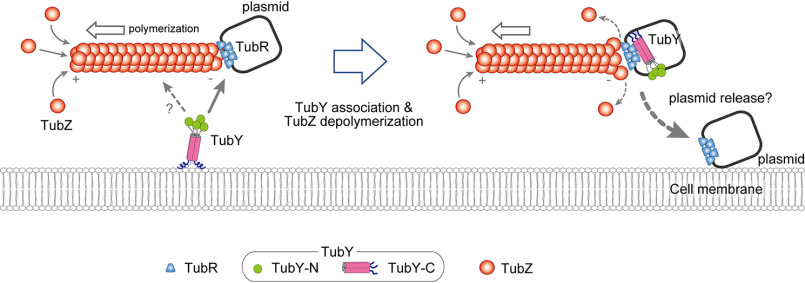
**Model for TubY-mediated plasmid release.** Hypothetical model for TubY-mediated plasmid release. In the absence of TubY, the plasmid is transported by the TubZ filament (10). When the segrosome at the minus-end of the filament approaches TubY attached to the cell membrane (*left*), TubY may dissociate from the membrane to interact with the TubR-centromere complex (*right*). TubY induces dissociation of the TubR-plasmid complex from the TubZ filament (16), delivering the plasmid to the daughter cell.

Based on the observation that TubZ filaments from *B. thuringiensis* seem to treadmill at the cell periphery, it has been speculated that the cell membrane is involved in detachment of the segrosome from the filaments (34, 43). These findings suggest that TubY and lipid membranes act as mediators in type III partition systems (Fig. 7). The TubR-*tubC* complex transported by the treadmilling TubZ filament encounters TubY at the membrane, detaches itself from the minus-end of the polymer, and associates with TubY, ultimately leading to plasmid partition. In this context, TubY is a key component of the partition machinery that serves to release the segrosome at a certain location within the cell.

## Experimental Procedures

### Protein preparation

The *tubY* gene of pBc10987 was cloned in-frame into pET28a using NdeI and NotI with an N-terminal histidine tag and an additional tobacco etch virus (TEV) protease recognition sequence. TubY and its mutants (BcYΔ: residues 1–182; BcY_mut_: BcY with R28A and K47A mutations; BcYN: 1–93; BcYC: 107–201) were expressed in BL21(DE3). Cells were suspended in a buffer containing 20 mm Tris (pH 8.0), 0.1 M NaCl, 5 mm β-mercaptoethanol and 3 M urea and lysed by ultra-sonication. Then the histidine-tagged BcY or its mutants were isolated using HisTrap (GE Healthcare). Denatured proteins were refolded by dialysis at 4 °C in a stepwise fashion (1, 0.5 and 0 M urea). After cleavage of the tag by the histidine-tagged TEV protease, the protein was loaded onto a HiTrap Q and Superdex 75 (GE Healthcare). CD spectroscopy confirmed that the purified proteins had secondary structures, and that the mutations of R28A and K47A did not affect folding. The gene encoding the TubY C-terminal domain (BcYCΔ; residues 107–182) was cloned into pET21. The protein was expressed in a soluble form in BL21(DE3) and purified from the bacterial extract by precipitation with 60% saturated ammonium sulfate, followed by HiTrap SP cation exchange chromatography (GE Healthcare). Selenomethionine-substituted BcYCΔ was expressed in BL21(DE3) by inhibition of the methionine biosynthetic pathway and purified as for the native protein (44). TubR was purified as reported previously (21).

### Crystallization, data collection, and structure determination

Se-substituted BcYCΔ protein was concentrated to 1.4 mm in a buffer containing 10 mm Tris (pH 8.0), 0.1 M NaCl and 3 mm DTT. Crystals were grown at 20 °C by sitting-drop vapor diffusion at 20 °C with a reservoir solution containing 0.1 M HEPES (pH 7.5), 0.2 M MgCl_2_ and 30% PEG400, and then frozen in a nitrogen stream at 95K.

All diffraction data were collected at the Photon Factory (Tsukuba, Japan). Diffraction images were processed with HKL2000 (22, 44). Initial phases were obtained by Se-SAD with the Phenix program AutoSol (45; Table 1). After automatic model building, the remaining residues were built manually in Coot (46). The structures were refined using Refmac (47). All figures were generated using PyMOL (48).

### DNA binding

DNA fragments of interest were generated by PCR with [5′-^32^P]-labeled primers. To identify the binding region of TubY or TubR, His-tag fused proteins were immobilized on Ni-NTA agarose beads (Qiagen). Bound proteins were mixed with a radioactive probe in 10 mm Tris-HCl (pH 8.0), 0.1 M KCl, 1 mm MgCl_2_, 0.05 mg/ml salmon sperm DNA and 0.1 mg/ml BSA, and incubated on ice for 30 min. Beads were washed with buffer containing 10 mm Tris-HCl (pH 8.0), 0.1 M KCl and 0.05% Tween20. The remaining radioactivity was counted in a scintillation counter. The protocol used for EMSA was described previously (21). Experiments were performed in triplicate.

### Hydroxyl radical footprinting

Hydroxyl radical footprinting was carried out as described previously (21, 49). Binding of TubR and BcY to the *pro2* region (nt 65321–65577) was analyzed by end-labeling the DNA fragment with [γ-^32^P]ATP. For footprinting experiments, *pro2* was cloned into pCR2.1 (Thermo Scientific) and digested with a restriction enzyme to create a single-end labeled DNA probe.

### Liposome pelleting assay

Phosphatidylethanolamine (PE) and phosphatidylglycerol (PG) from chicken egg were purchased from Sigma and prepared as described previously (41). PE and PG were mixed at a ratio of 7:3. Dried lipids were solubilized in 20 mm HEPES (pH 7.0) and 0.1 M NaCl, and sonicated for 20 min until the solution became clear.

For pelleting experiments, 0.5 mg/ml BcY or BcYΔ and 2.5 mg/ml liposomes were mixed and incubated for 10 min at 20 °C. The mixture was then centrifuged using a Beckman TLA100.3 rotor at 80,000 rpm for 20 min. Pellets were analyzed by SDS-PAGE with Coomassie Brilliant Blue staining. Experiments were performed in triplicate.

### CD measurements

The CD spectra of the peptide encompassing the C-terminal tail of BcY (BcY_tail_; residues 183–201; CS Bio) were collected between 195 and 260 nm at 20 °C using a JASCO J-720W spectropolarimeter with a 0.1 cm pathlength quartz cuvette. Samples at a concentration of 0.1 mg/ml in the presence or absence of 2.5 mg/ml phospholipids in a buffer containing 20 mm HEPES (pH 7.0) and 0.1 M NaCl were scanned four times with a step size of 0.5 nm and averaged.

### Fluorescence microscopy

The genes encoding *tubY* and its mutants were inserted into the N-terminal GFP fusion vector pSG1729 using the BamHI and XhoI sites and transformed into *B. subtilis* 168 as described previously (50). Overnight cultures were diluted 1:100 in fresh LB supplemented with 250 μg/ml spectinomycin and grown to exponential phase at 30 °C. Expression of GFP-fused protein was induced with 0.75% xylose. Cells were washed three times with PBS, immobilized on microscope slides covered with 1% agarose in PBS and visualized under a confocal laser scanning microscope (Leica TCS SP8, Leica Microsystems).

## Data availability

Crystallographic data and coordinates were deposited in Protein Data Bank with the accession number of 7C7Y.

## Supplementary Material

Supporting Information
