# Supplementary material for: The C-terminal region of the plasmid partitioning protein TubY is a tetramer that can bind membranes and DNA
Source: J Biol Chem. 2020 Oct 22;295(51):17770–80. doi: 10.1074/jbc.RA120.014705 (PMC7762940; doi:10.1074/jbc.RA120.014705)
Supplement: Supporting Information [file supp_295_51_17770__index.html]

The C-terminal region of the plasmid partitioning protein TubY is a tetramer that can bind membranes and DNA — Crystal structure of TubY tetramerization domain — The C-terminal region of the plasmid partitioning protein TubY is a tetramer that can bind membranes and DNA — Crystal Structure of TubY Tetramerization Domain — Supporting Information 

# The C-terminal region of the plasmid partitioning protein TubY is a tetramer that can bind membranes and DNA

## Supporting Information

- Supporting Information (to be published online) - Supplementary Figure
