# Supplementary material for: The C-terminal region of the plasmid partitioning protein TubY is a tetramer that can bind membranes and DNA
Source: J Biol Chem. 2020 Oct 22;295(51):17770–80. doi: 10.1074/jbc.RA120.014705 (PMC7762940; doi:10.1074/jbc.RA120.014705)
Supplement: Supporting Information [file supp_RA120.014705_161335_2_supp_610650_qz2dy6.pdf]

# Supporting Information

# The C-terminal region of the plasmid partitioning protein TubY is a tetramer that can bind membranes and DNA

**Ikuko Hayashi**

Department of Medical Life Science, Yokohama City University, 1-7-29 Suehiro, Tsurumi, Yokohama, Kanagawa 230-0045, Japan

Correspondence should be addressed: Tel.: 81-45-508-7214; Fax: 81-45-508-7361; E-mail: ihay@yokohama-cu.ac.jp

## Supplementary References

1. Watanabe, S., Kita, A., Kobayashi, K. & Miki K (2008) Crystal structure of the [2Fe-2S] oxidative-stress sensor SoxR bound to DNA. *Proc Natl Acad Sci U S A*. **105**, 4121-4126
2. Schumacher, M.A., Chinnam, N.B., Cuthbert, B., Tonthat, N.K. & Whitfill, T. (2015) Structures of regulatory machinery reveal novel molecular mechanisms controlling B. subtilis nitrogen homeostasis. *Genes Dev*. **29**, 451-464
3. Hayashi, I., Oda, T., Sato, M., & Fuchigami, S. (2018) Cooperative DNA Binding of the Plasmid Partitioning Protein TubR from the *Bacillus cereus* pXO1 Plasmid. *J. Mol. Biol*. **430**, 5015-5028

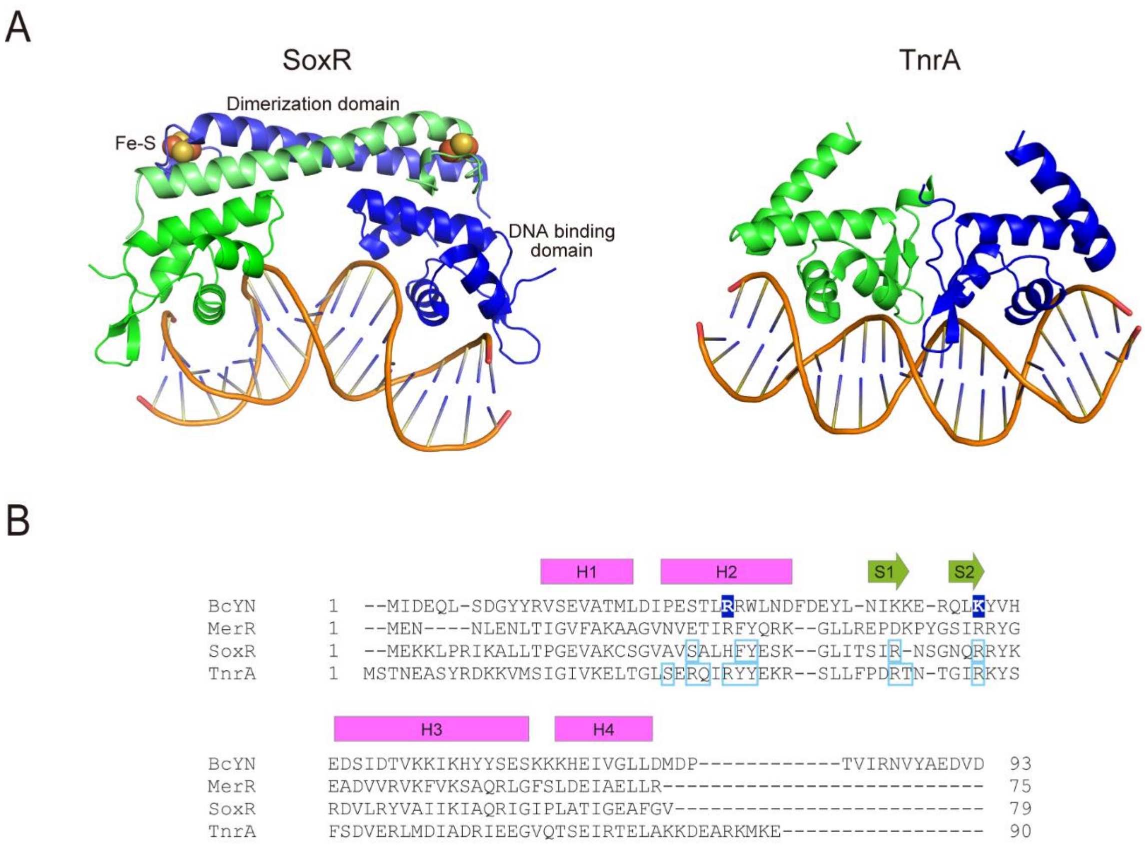


**Supplementary Figure S1. BcYN and the MerR family proteins**

(A) (left) SoxR bound to DNA (PDB code: 2ZHG[1]). SoxR is a typical MerR family protein that contains an antiparallel dimerization domain at its C terminus. DNA-binding domains of each monomer are shown in blue and green, and the C-terminal coiled-coils are shown in light green and sky blue. The [2Fe-2S] cluster, which activates SoxR, is shown in sphere representation. (right) TnrA bound to DNA (4R24[2]). TnrA contains a single DNA-binding domain and forms a dimer to associate with DNA. The two DNA-binding domains are shown in blue and green.

(B) Sequence alignment of HTH motifs in BcYN, MerR, SoxR and TnrA. Secondary structural elements are labeled above the sequences. Residues boxed in cyan are involved in DNA contact[1,2]. Residues mutated in this study (R28 and K47) are highlighted in blue.

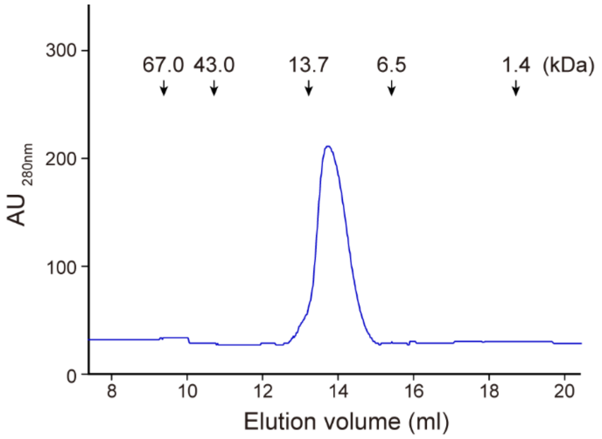


**Supplementary Figure S2. BcYN is a monomer**

Gel-filtration analysis of BcYN. Purified BcYN was analyzed by a Superdex75 10/300 gel-filtration column (GE Healthcare) equilibrated with 10 mM Tris (pH 8.0), 0.1 M NaCl and 1 mM DTT. The elution volumes of molecular mass standards are indicated by arrows. BcYN (theoretical molecular weight: 11.4 kDa) is a monomer.

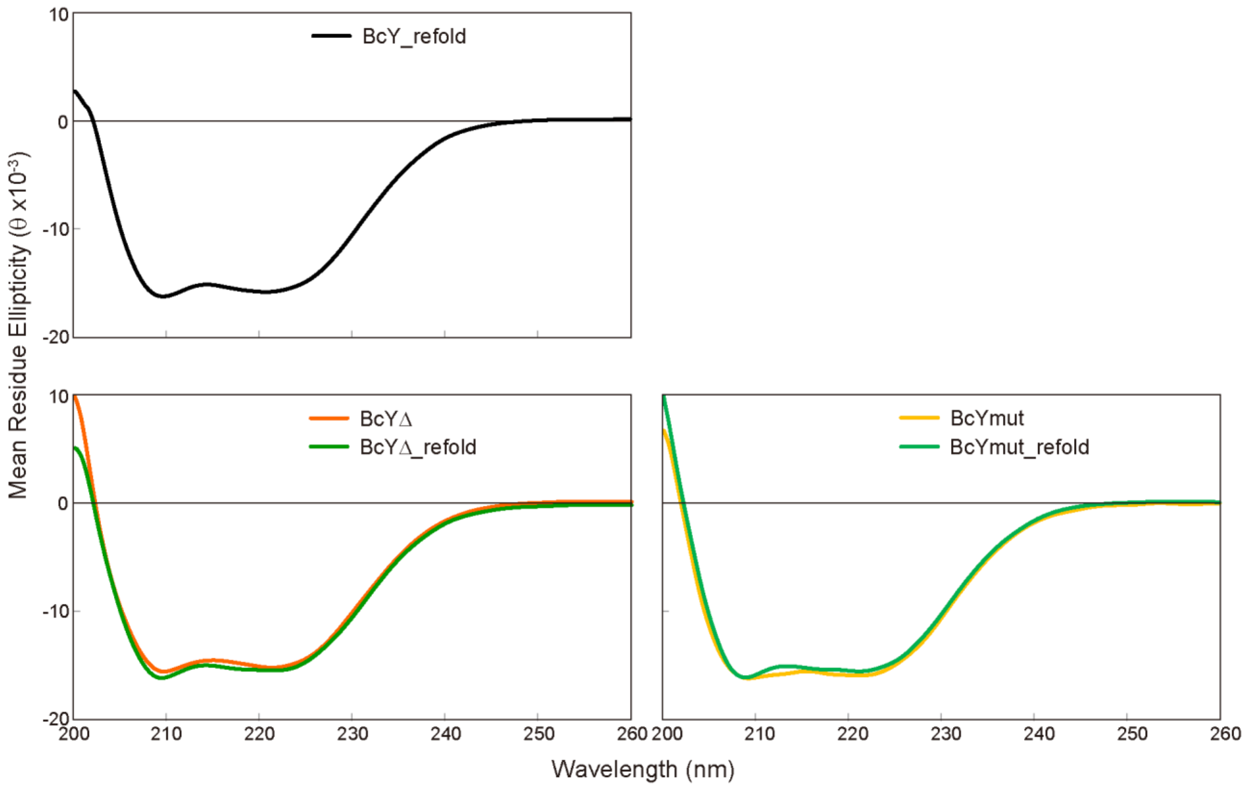


**Supplementary Figure S3. CD spectra of BcY and its mutants**

(Top) Refolded BcY. (Bottom left) BcYΔ (orange: soluble BcYΔ; green: refolded BcYΔ). (Bottom right) $BcY_{mut}$ (yellow: soluble $BcY_{mut}$; light green: refolded $BcY_{mut}$). Refolded proteins were used in this study.

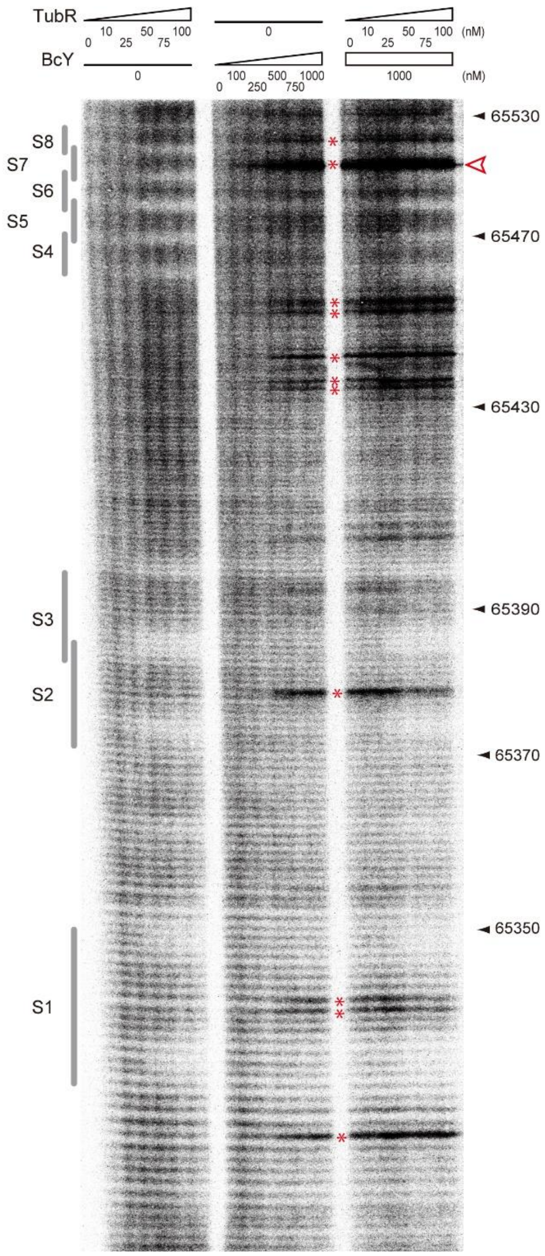


**Supplementary Figure S4. Centromere binding of TubR and BcY**

Hydroxyl radical footprint analysis of the *pro2* region. DNA probe was radiolabeled on the sense strand. The amounts of TubR and BcY used (in nM) are indicated above the lanes. The numbers on the right-hand side show the location on the pBc10987 plasmid. Gray bars on the left-hand side indicate the regions of the TubR recognition sites (S1-S8)[3]. Hypersensitive sites induced by BcY are marked by red asterisks. At higher concentrations of TubR (> 50 nM), BcY protects the TubR-binding region (S1-S3) together with TubR. Note that, while in the presence of BcY the S7 TubR-binsing site is sensitive to hydroxyl radical on the sense strand, the hypersensitive degree at S7 is decreased on the anti-sense strand when the dose of TubR was increased (red arrow; Fig. 4B), implying that BcY induces a kink at S7 but protects the antisense strand in the presence of TubR.

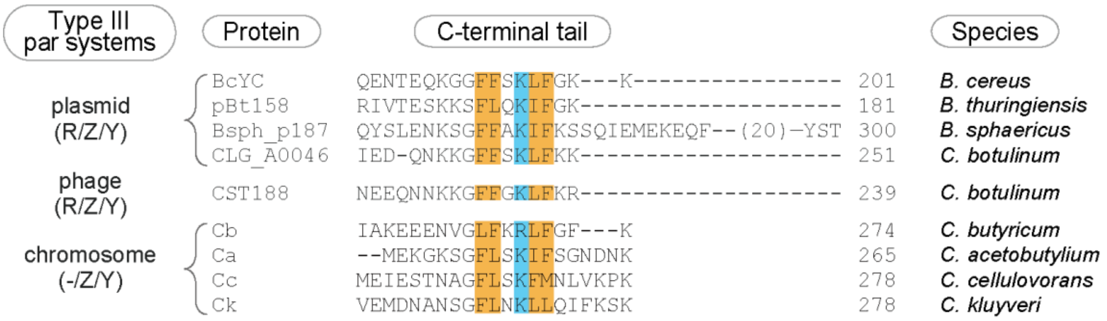


**Supplementary Figure S5. The C terminus of TubY**

An alignment of the TubY tail from *Bacillus* and *Clostridium* species. The C-terminal tail of TubY contains a conserved sequence motif. Conserved hydrophobic residues are highlighted in orange; conserved basic residues in cyan. Presence or absence of *tubR* (R) in type III *par* systems are shown in brackets. *Clostridial* TubY sequences from phage and chromosomes are also shown. Cb: *C. butyricum* (NCBI Reference Sequence ID: WP_003407033), Ca: *C. acetobutylium* (WP_010966727), Cc: *C. cellulovorans* (WP_010074309), Ck: *C. kluyveri* (EDK32625).
